# Meta-Analysis of Gene Expression Signatures Reveals Hidden Links among Diverse Biological Processes in Arabidopsis

**DOI:** 10.1371/journal.pone.0108567

**Published:** 2014-11-14

**Authors:** Liming Lai, Steven X. Ge

**Affiliations:** Department of Mathematics and Statistics, South Dakota State University, Brookings, South Dakota, United States of America; University of Jaén, Spain

## Abstract

The model plant Arabidopsis has been well-studied using high-throughput genomics technologies, which usually generate lists of differentially expressed genes under various conditions. Our group recently collected 1065 gene lists from 397 gene expression studies as a knowledgebase for pathway analysis. Here we systematically analyzed these gene lists by computing overlaps in all-vs.-all comparisons. We identified 16,261 statistically significant overlaps, represented by an undirected network in which nodes correspond to gene lists and edges indicate significant overlaps. The network highlights the correlation across the gene expression signatures of the diverse biological processes. We also partitioned the main network into 20 sub-networks, representing groups of highly similar expression signatures. These are common sets of genes that were co-regulated under different treatments or conditions and are often related to specific biological themes. Overall, our result suggests that diverse gene expression signatures are highly interconnected in a modular fashion.

## Introduction

Because of its small genome size, *Arabidopsis* thaliana has been a valuable model system for genetic mapping, sequencing and gene expression analysis [1]. Until March 2013, 1787 studies on gene expression of *Arabidopsis* were indexed in Gene Expression Omnibus (GEO) website in National Center for Biotechnology Information (NCBI) [2]. These studies investigated various biological processes by monitoring the gene expression level using the high-throughput genomics technologies such as DNA microarrays and RNA sequencing. The results were usually a set of genes associated with particular biological processes based on different experimental designs. Even though DNA microarrays suffer from noise and reproducibility issues [3], we believe that many of the noise could be filtered out by statistical analysis and that there are significant associations among these numerous results, or common modules in the transcriptional program.

Some studies have showed the relationships among gene lists in different species. Most researchers analyzed these gene lists using methodology of meta-analysis [4]–[7], which combines the results of studies that address a set of related research hypotheses, focusing on a special individual topic such as cancer or special treatment [8]. Several databases of gene lists have been created, such as L2L [9], LOLA [10], and MSigDB [11]. An network-based method was developed by Ge [12] to define associations among a large number of gene sets in human. Associations are defined as statistically significant overlaps between two gene lists. The method was applied successfully to a large number of human gene lists [12], and identified molecular links among diverse biological processes.

In this study, we used the methodology in [12] to analyze a set of *Arabidopsis* gene lists identified by genome wide expression studies. These lists were collected for AraPath [13], an *Arabidopsis* gene lists database we created recently. The objective was to systematically evaluate relationships among the gene lists and interpret the relationships. This process provides not only a new tool to uncover hidden links among vast amounts of gene lists, but a quantitative measure to describe the global gene expression of the *Arabidopsis* system under diverse conditions.

## Materials and Methods

Data in this study was extracted from the AraPath [13], which is a gene lists database in *Arabidopsis* we created (Availability: http://bioinformatics.sdstate.edu/arapath/). As part of the database, the data contains a total of 1,065 co-expression gene lists, which were manually retrieved from published papers linked to GEO [2] before February, 2011.

Methodology of the analysis includes four steps. Step 1 is to evaluate overlapping genes among the 1,065 gene lists. A Perl programs was written to evaluate overlapping genes between all 566,580 pairs of lists. An overlap refers to a pair of gene lists, which has at least two common genes. And overlaps from the same paper were considered trivial and were removed. Because there are too much overlaps and microarray experiments tends to produce noisy data, we selected significant overlaps using stringent threshold. Step 2 computes p-values and q-values to identify significant overlaps. Based on the Hypergeometric distribution, we first calculate the likelihood (p-value) of observing the number of overlapping genes if these two gene lists are randomly drawn without replacement from a collection of 28,024 unique genes in terms of R program [14] we compiled. Then, p-values were translated into q-values based on the false discovery rate (FDR) [15] to correct that for multiple testing. Overlaps with very small q-value were significant overlaps. In this case, significant overlaps were identified with a q-value  = 5.0E-9 as a cutoff. In step 3, network of significant overlaps was constructed based on outputs of the step 2 using Cytoscape[16]. Because this network includes too many nodes and edges, we need to further break the big clusters into smaller subclusters. In step 4, There are many algorithms that could decompose large networks into small, densely connected subnetworks such as those in [17], [18]. We chose a simply algorithm that is available as a plug-in to Cytoscape. MCODE [19] is used to identify interconnected sub-networks and their clusters within the network of the step 3. To generally find locally dense regions (or clusters) of a graph is based on the clustering coefficient [19], *C_i_*, which measures “clique” of the neighborhood of a vertex: *C_i_ = 2n/k_i_ (k_i_ – 1)*, where *k_i_* is the vertex size of the neighborhood of vertex *i, n* is the number of edges in the neighborhood. According to the MCODE algorithm [19], however, clustering the main network into sub-networks is by means of vertex weighting, which is to weight all vertices based on their local network density using the highest *k*-core of the vertex neighborhood rather than the clustering coefficient *C_i_*. A *k*-core is a graph of minimal degree *k*. The highest *k*-core of a graph is the central most densely connected sub-graph. Given a highly connected vertex, in a dense region of a graph, *v* may be connected to many vertices of degree one. These low degree vertices do not interconnect within the neighborhood of *v* and thus would reduce the clustering coefficient, but not the core-clustering coefficient (for detailed information about the MCODE algorithms, see the paper [19]). Here we created the sub-networks and found the modules and clusters using MCODE algorithms based on the following parameters: Node Score Cutoff  = 0.15; k-core  = 2; Degree Cutoff  = 2; Max. Depth  = 100. The DAVID web site [20], [21] was applied to analyze the most significant functions of most frequently shared genes in each of sub-networks.

## Results

**Figure 1:**
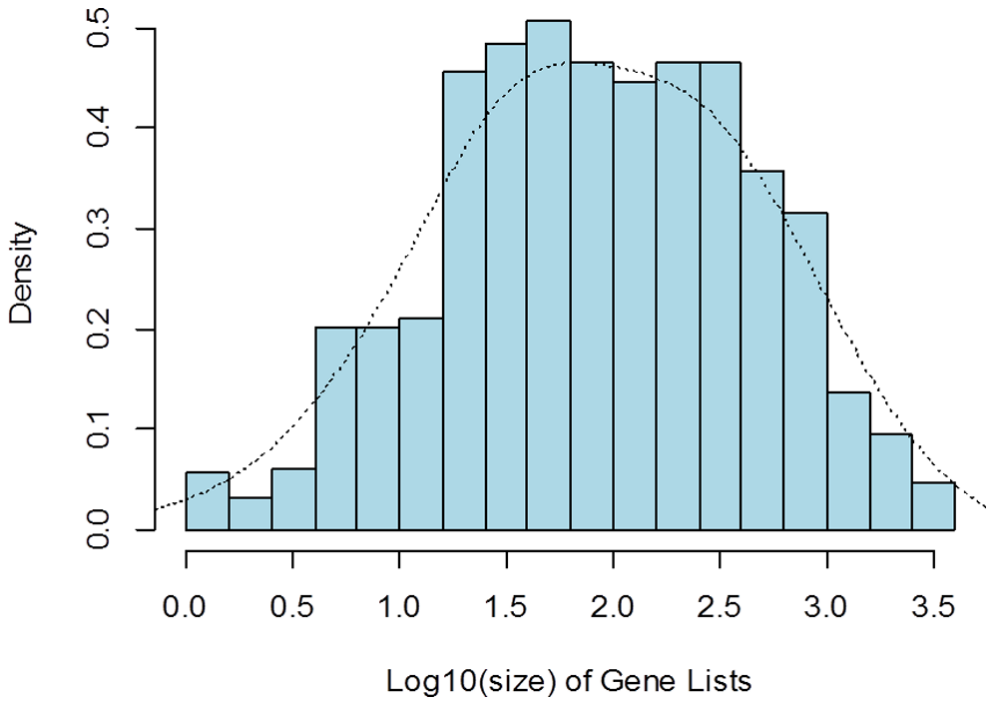
Histogram of log10 scale of size of gene lists.

A total of 1,065 gene lists were analyzed in this study. They include 277,349 gene entries corresponding to 28,024 unique genes. The average size of these gene lists is 87 genes, ranging from 1 to 2,952 genes. Its distribution is close to normality on a log10 scale (Figure 1). The results of analysis of the data are as follows.

### Significant overlaps

By comparing all pairs of 1,065 gene lists using the Perl program, 16,261 significant overlaps were identified from a total of 192,642 overlaps. Based on the Hyper-geometric distribution, the probabilities (p-values) of observing the number of overlapping genes or more were first calculated if these two gene lists were randomly drawn without the replacement from a collection of 28,024 unique genes. The p-values were translated into q-values according to the false discovery rate (FDR) [15] to correct for multiple testing. Overlaps from the same paper were considered trivial and were removed. With a q-value  = 5×10^−^as a conservative cutoff, 16,261 significant overlaps were identified.

### Main network

**Figure 2:**
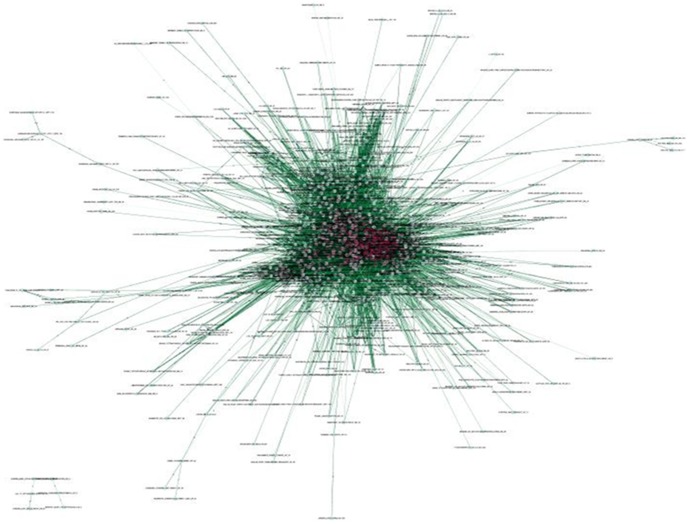
The main network created by Cytoscape. Node  =  name of gene list. Node Color  =  MCODE_Scores from small to large and corresponds to color from light green to dark red. Edge Color  =  p-values from large to small and corresponds to color from grey to dark green.

The 16,261 significant overlaps are represented as an undirected network (Figure 2). In the network, nodes correspond to gene lists and edges indicate number of overlapping genes between two nodes within significant overlaps. This network highlights the correlation across gene expression signatures of diverse biological processes. It, thus, constitutes a “molecular signature map” in which the individual perturbations are placed in the context defined by others. This is a highly connected network with an average of 20.10 connections per gene list. The 809 nodes (75.96% of the 1,065 gene lists) and 16,261 edges are connected to a dominant main network. Most nodes are connected to a small number of gene lists. The network shows some different colors “cliques”, which are some of the most connected graphs in terms of a vertex-weighting scheme based on the highest *k*-core of the vertex neighborhood. They are intuitively denser links within some neighborhoods.

### Modules

To further explore these neighborhoods, we used the MCODE algorithm [19] to decompose the network into a total of 20 sub-networks. Of them, nine modules were further analyzed and their clustered information was shown in Table 1. The others were ignored because they are much lower score of density (less than 1.6) and have few nodes and edges (less than 6). The nine sub-networks are highly interconnected, suggesting that those genes are involved in common metabolic pathways or interact with each other under similar biological perturbations. The first three sub-networks are described in the following section. The remaining six sub-networks description and all the sub-networks figures and their composite outcomes tables are shown in Figures S1–S9 and Tables S1–S9 in File S1.

**Table 1:** Summary of nine modules consisting of highly interconnected gene lists.

ID	Score Density†	#Nodes	#Edges	Unique genes	Shared genes*	Highest frequency	Most significantly enriched GO Term*	P-value*
1	21.065	46	969	10581	256	35	response to chitin	1.70E-38
2	9.907	54	535	12163	168	20	plastid thylakoid membrane	1.80E-59
3	9.545	33	315	9501	124	17	response to chitin	8.80E-13
4	9.062	48	435	13930	155	18	response to auxin stimulus	9.00E-10
5	2.559	34	87	9751	109	10	cell wall	5.30E-11
6	2.111	9	19	2565	34	6	External encapsulating structure organization	5.40E-07
7	2	15	30	6725	53	8	glycoside biosynthetic process	2.00E-05
8	2	6	12	5212	66	6	membrane-enclosed lumen	6.00E-14
9	1.846	13	24	3567	23	6	response to abiotic stimulus	6.80E-08
T	_	258	2426	_	988	_	_	_

Note: †Score Density  =  #Edges/#Nodes. *Most significantly enriched GO Term and p-value are the results of analysis of the.

DAVID in terms of the most frequently shared genes (i.e. Shared genes in Table 1) in each sub-network.

### Sub-network 1

**Figure 3:**
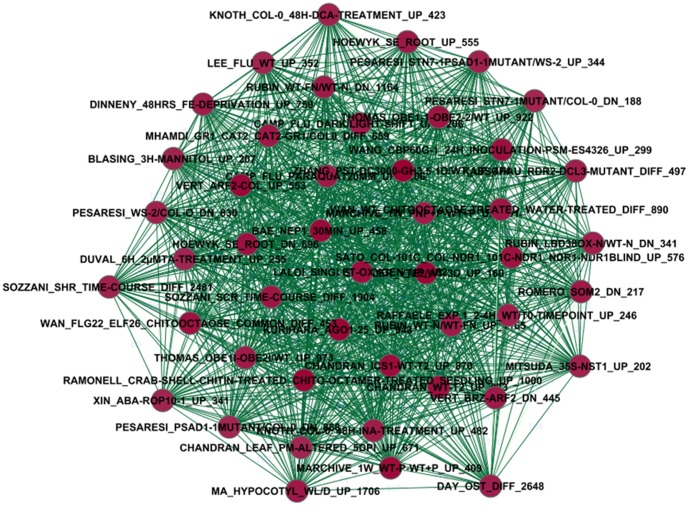
Sub-network 1 corresponding to the module 1/cluster 1 (see note in **Figure 2** for meanings of node and edge and their color).

The sub-network 1 includes 46 nodes and 969 edges (Figure 3). The score of cluster density is 21.065, which is the highest score among nine sub-networks. This indicates it is the most densely connected. In Figure 3, the dark red nodes represent higher network density based on MCODE [19]. Dark green edges represent very small p-values. There are 31 nodes that represent up-regulated, eight down-regulated, and seven differently regulated. Most gene lists (67.39%) involving up-regulated nodes are related to seven biological themes and 25 treatments or conditions. Table S1 in File S1 shows its composite outcomes. There are 46 gene lists and 256 frequently shared genes identified in this sub-network. They are regulated by 32 treatments or conditions from 32 publications related to 11 biological themes involving development, metabolism, disease, yield, function, genome analysis, immune, pathogen, mechanism, energy, virus, and photosynthesis in *Arabidopsis*. The top 10 most frequently shared genes with their gene descriptions were specifically listed corresponding to different gene lists, different biological themes, and treatments or conditions. For example, gene AT4G14365 (“putative E3 ubiquitin-protein ligase XBAT34”) has the highest frequency of 35, which indicates it is the most active gene because it is regulated simultaneously under 35 gene lists in sub-network 1, namely, the gene connects directly 35 gene lists.

The most significant function of sub-network 1 is biological process in response to chitin (i.e. the most enriched term corresponding to the most frequently shared genes in sub-network 1 is “response to chitin”) based on results of analysis of DAVID (Table 1). This indicates sub-network 1 is specifically associated with the chitin signaling pathway rather than by random chance. The other significant functions of sub-network 1 are responding to carbohydrate stimulus, organic substance, defense response, and bacterium based on the analysis of DAVID with a cutoff of 1.60×10^−16^ p-value. This suggests that sub-network 1 involves multiple signaling pathways.

### Sub-network 2

**Figure 4:**
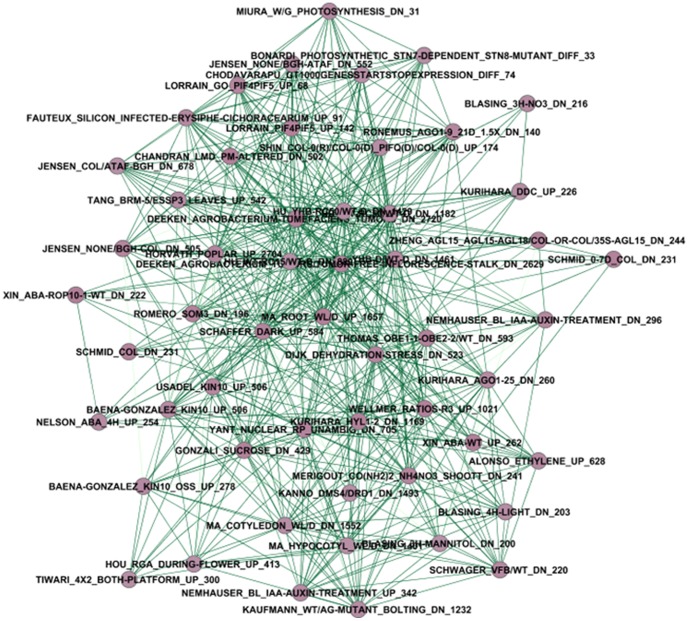
Sub-network 2 corresponding to the module 2/cluster 2 (see note in **Figure 2** for meanings of node and edge and their color).

Sub-network 2 is shown in Figure 4 and Table S2 in File S1. It includes 54 nodes (gene lists) and 168 frequently shared genes, which are regulated under 38 different treatments or conditions from 38 publications related to 10 biological themes. The score of cluster density is 9.907. There are 17 nodes to be up-regulated, 34 to be down-regulated, and three to be differently regulated. Most gene lists (62.96%) involving down-regulated nodes are related to nine biological themes and 23 treatments or conditions. Compared to sub-network 1, sub-network 2 has a lower cluster density score with even more treatments or conditions. Nine themes in sub-network 2 are common with sub-network 1: development, disease, function, genome analysis, mechanism, metabolism, photosynthesis, virus, and yield. This indicates the two sub-networks have relationships linked by same themes. No gene is common between the 256 frequently shared genes in sub-network 1 and the 168 frequently shared genes in sub-network 2. The two sub-networks have relatively independent functions. The most significant function of sub-network 2 is biological process of plastid thylakoid membrane based on results of DAVID (Table 1), suggesting that sub-network 2 is specifically associated with plastid thylakoid membrane, i.e. the lipid bilayer membrane of any thylakoid within a plastid. The other significant functions of sub-network 2 are response to chloroplast thylakoid membrane, thylakoid membrane, plastid thylakoid, and chloroplast thylakoid based on DAVID with cut-off p-value of 6.8×10^−58^. The top 10 most frequently shared genes with their gene descriptions corresponding to gene lists, biological themes, and treatments or conditions were specifically listed in Table S2 in File S1. Gene AT4G27030 (fatty acid desaturase A), for example, has the highest frequency of 20, indicating it is the most active gene in sub-network 2.

### Sub-network 3

**Figure 5:**
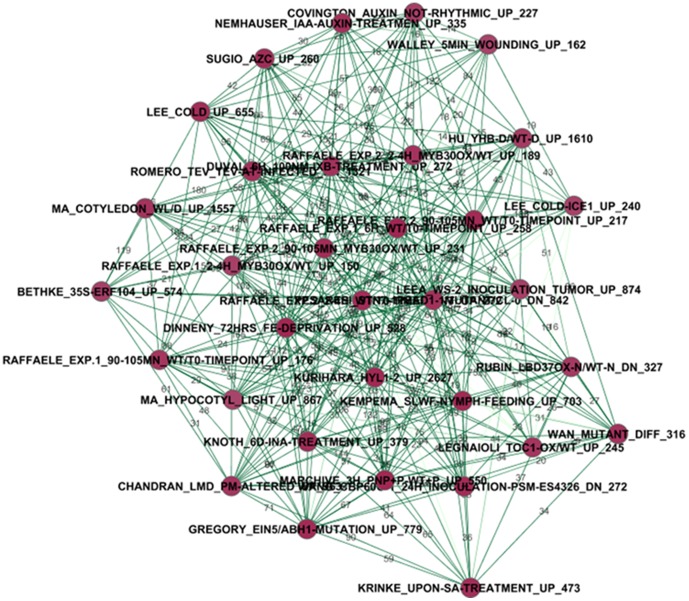
Sub-network 3 corresponding to the module 3/cluster 3 (see note in **Figure 2** for meanings of node and edge and their color. edge label  =  number of overlapping genes between two nodes).

The sub-network 3 includes 33 nodes (gene lists) and 124 most frequently shared genes (Figure 5 and Table S3 in File S1). There are 28 nodes to be up-regulated, four to be down-regulated, and one to be differently regulated. Most gene lists (84.85%) involving up-regulated nodes are related to nine biological themes and 20 treatments or conditions. By contrast to sub-networks 1 and 2, sub-network 3 is smaller in size, has a lower cluster density score, and less treatments or conditions for gene lists. Sub-network 3 are regulated by 25 treatments or conditions from 25 publications associated with 11 biological themes. Nine themes in sub-network 3 are common with sub-network 1: development, disease, energy, function, immune, mechanism, metabolism, photosynthesis, and virus. There are seven common themes (development, disease, function, mechanism, metabolism, photosynthesis, and virus) between sub-networks 3 and 2. These indicate sub-networks 3 and 1 or 2 have relationships linked by the same themes.

The top 10 most frequently shared genes with their gene descriptions corresponding to each gene list in sub-network 3 are specifically listed in Table S3 in File S1. Gene AT2G18690 has the highest frequency at 17, indicating it is the most active genes in sub-network 3. Other shared genes in sub-network 3 have lower frequency, which means they are less active than those in sub-networks 1 and 2. There is no common gene between the 168 most frequently shared genes in sub-network 2 and the 124 most frequently shared genes in sub-network 3. However, there are 72 genes of intersection between sub-networks 1 and 3. This indicates sub-networks 2 and 3 have relatively independent functions and sub-networks 1 and 3 have dependent relationship linked by the common shared genes. The most significant function of sub-network 3 is biological process in response to chitin based on results of analysis of DAVID (Table 1). It is specifically associated with the chitin signaling pathway, which is the same as sub-network 1 but different from sub-networks 2.

## Discussion

The gene lists in our data are highly connected. Out of the 1,065 gene lists, 75.96% are connected in the main network in which many seemingly unrelated stimuli/perturbation may activate or deactivate the same molecular pathways. All the gene lists within each sub-network are highly connected by the most frequently shared genes. For example, in sub-network 8 (Figure S8 and Table S8 in File S1), AT1G56110 (“homolog of nucleolar protein NOP56”), AT3G05060 (“putative SAR DNA-binding protein”), and AT3G44750 (HDA3 histone deacetylase HDT1) are regulated by six different treatments or conditions from five publications corresponding to five special biological themes, which involves reproduction, photosynthesis, metabolism, development, and yield in *Arabidopsis* (Table S8 in File S1). And the most enriched term in sub-network 8 is membrane-enclosed lumen based on functional analysis of DAVID (Table 1). Therefore, the three genes not only connect the six gene lists, but have multiple functions by which we could propose a hypotheses that the three genes' interaction controls the reproduction, photosynthesis, metabolism etc. Furthermore, the three genes associated with the rapidly proliferating nature of the endosperm at 4 DA, with similar expression patterns to the early endosperm markers SUC5, PHE1, FWA, and FIS2 [22], were regulated by interploidy crosses, fis1X2x crosses at 5 DAP (two biological replicates of each), and unfertilized msi1 siliques at 7 DAF [23], kin10, starvation conditions, and sugar availability increase [24], sucrose [25], and 4h-carbon fixcation [26]. These all became the organized links among the six gene lists because they associated with the three genes.

However,there are significant differences among the nine sub-networks. Based on Table 1, sub-networks 1–5 have more nodes than sub-networks 6–9. This does not mean the genes in sub-network 6–9 are less important than those in sub-networks 1–5. Generally, the most significantly enriched GO terms in sub-networks 1–9 are different except for sub-networks 1 and 3, which have the same theme “response to chitin”. The second, third, and fourth most significantly enriched GO Terms in sub-network 1 are response to carbohydrate stimulus, organic substance, and defense response, respectively, which are different from those in sub-network 3. This indicates there are significantly different functions in sub-networks 1–9. Furthermore, “cliques” of all sub-networks are different in the whole network with 16,261 significant overlaps. These “cliques” are some of the most connected graphs in the NETWORK in terms of a vertex-weighting scheme based on the highest *k*-core of the vertex neighborhood. Therefore, they specify different meanings and information. Finally, all the most frequently shared genes of the sub-networks are different. For instance, sub-network 7 has the second smallest score of 2, with 15 nodes, and 30 edges, but possesses 53 most frequently shared genes, which are completely different from that of sub-network 1.

The most frequently shared genes are the strongest links within each of nine sub-networks and provide genomics research with important insights. They are the most important results we found in this study. For example, the gene AT5G39670 (“putative calcium-binding protein CML45”, function as calcium ion binding) has the second highest frequency of 34 in the sub-network 1 (Table S1 in File S1). Its function as calcium ion binding indicates calcium-binding proteins participate in calcium cell signaling pathways by binding to Ca^2+^. These proteins are expressed in many cell types during various growth stages in plants, and contribute to all aspects of the cell's functioning [24]. In the present study, we found that this gene responded to 24 treatments or conditions such as necrosis-ethylene, diurnal cycle, salicylic acid, iron deprivation, etc. according to various reports. Furthermore, this gene mediates 10 crucial biological themes: immune, yield, disease, development, metabolism, function, photosynthesis, pathogen, energy, and virus. Also, the gene belongs to 24 up-regulated gene lists, seven down-regulated gene lists, and three different-regulated gene lists. In most cases, this gene is up-regulated, indicating that when the experimental treatments listed above were applied, there was an increased expression of the gene AT5G39670. However, some treatments or stimuli may cause a decreased expression of the above gene in order to protect its cells. Therefore, AT5G39670 is the second most active gene and second strongest link in sub-network 1. Similarly, gene AT4G14365 (“XBAT34”, molecular functions: protein binding and zinc ion binding) is the strongest link as it is related to 35 gene lists in sub-network 1. Gene AT3G50930 (“BCS1”, molecular functions: ATP binding and ATPase activity) is the third strongest link because it is associated with 33 gene lists in sub-network 1. Also, the most frequently shared genes are significantly different in these nine sub-networks. For instance, there are 124 most frequently shared genes in sub-network 3 and 155 most frequently shared genes in sub-network 4. However, there are only two common genes between these two sub-networks. These important results and their biological mechanisms need to be further addressed.

The results from this study, summarized as a molecular signature map, provide key insights into the underlying connections of diverse perturbations. More importantly, compared to previous reports that focused on specific themes, this study explored and established the hidden links among the gene lists on a global scale in *Arabidopsis*. These sub-networks will provide new putative gene targets for future research. For example, sub-network 4 shows that the top three genes AT1G74670, AT1G04240, and AT1G69530 are down-regulated by bioactive gibberellins corresponding to the gene list ZENTELLA_DEX_VECTOR_DN_244 (Table S4 in File S1). This information provides some possible clues for future research regarding the mechanism of the regulation of plant growth by plant hormone gibberellins. Another example is gene AT4G27030, which has the highest frequency of 20 in sub-network 2. This gene is regulated by 14 treatments or conditions such as agrobacterium tumefaciens, kin10 and kin 11, dark, far-red light, etc. (Table S2 in File S1). This gene could be used to genetically modify crops for new and useful functions.

## Conclusions

There are hidden links among the gene lists from the published papers concerning *Arabidopsis*. After performing systematic overlap analysis, we created 10 networks, including network and sub-networks 1–9 where there are a number of links among gene lists. Many seemingly unrelated stimuli/perturbation may activate or deactivate the same molecular pathways. These links are actually a set of overlapping genes. Of them, a total of 988 most frequently shared genes were identified from each sub-network. These genes are regulated by multiple treatments or conditions from different gene lists and related to different biological themes based on their sub-networks. They construct more active (stronger) links among the gene lists in our data.

Compared to previous reports focusing on specific themes, this study explored and established hidden links among the gene lists on a global scale in *Arabidopsis*. These results provide significant information about target genes or models for future research. In the future, it will be necessary for us to extend gene lists and develop more effective analysis methods to further explain the booming gene lists of microarray data.

## Supporting Information Legends

File S1 Fig. S1. Sub-network 1 corresponding to cluster 1. Node  =  name of gene list. Node Color  =  MCODE_Scores from small to large and corresponds to color from light green to dark red. Edge Color  =  p-values from large to small and corresponds to color from grey to dark green. Fig. S2. Sub-network 2 corresponding to cluster 2. Fig. S3. Sub-network 3 corresponding to cluster 3. Fig. S4. The sub-network 4 correspoding to cluster 4. Fig. S5. The sub-network 5 corresponding to Cluster 5. Fig. S6. The sub-network 6 corresponding to Cluster 6. Fig. S7. The sub-network 7 corresponding to Cluster 7. Fig. S8. The sub-network 8 corresponding to Cluster 8. Fig. S9. The sub-network 9 corresponding to Cluster 9. Table S1. Results of sub-network 1 corresponding to cluster 1. Table S2. Results of sub-network 2 corresponding to cluster 2. Table S3. Results of sub-network 3 corresponding to cluster 3. Table S4. Results of sub-network 4 corresponding to cluster 4. Table S5. Results of sub-network 5 corresponding to cluster 5. Table S6. Results of sub-network 6 corresponding to cluster 6. Table S7. Results of sub-network 7 corresponding to cluster 7. Table S8. Results of sub-network 8 corresponding to cluster 8. Table S9. Results of sub-network 9 corresponding to cluster 9.
(DOCX)

Checklist S1 (DOC)
