# Supplementary material for: Meta-Analysis of Gene Expression Signatures Reveals Hidden Links among Diverse Biological Processes in Arabidopsis
Source: PLoS One. 2014 Nov 14;9(11):e108567. doi: 10.1371/journal.pone.0108567 (PMC4232243; doi:10.1371/journal.pone.0108567)
Supplement: File S1 — Fig. S1. Sub-network 1 corresponding to cluster 1. Node = name of gene list. Node Color = MCODE_Scores from small to large and corresponds to color from light green to dark red. Edge Color = p-values from large to small and corresponds to color from grey to dark green. Fig. S2. Sub-network 2 corresponding to cluster 2. Fig. S3. Sub-network 3 corresponding to cluster 3. Fig. S4. The sub-network 4 correspoding to cluster 4. Fig. S5. The sub-network 5 corresponding to Cluster 5. Fig. S6. The sub-network 6 corresponding to Cluster 6. Fig. S7. The sub-network 7 corresponding to Cluster 7. Fig. S8. The sub-network 8 corresponding to Cluster 8. Fig. S9. The sub-network 9 corresponding to Cluster 9. Table S1. Results of sub-network 1 corresponding to cluster 1. Table S2. Results of sub-network 2 corresponding to cluster 2. Table S3. Results of sub-network 3 corresponding to cluster 3. Table S4. Results of sub-network 4 corresponding to cluster 4. Table S5. Results of sub-network 5 corresponding to cluster 5. Table S6. Results of sub-network 6 corresponding to cluster 6. Table S7. Results of sub-network 7 corresponding to cluster 7. Table S8. Results of sub-network 8 corresponding to cluster 8. Table S9. Results of sub-network 9 corresponding to cluster 9. (DOCX) [file pone.0108567.s001.docx]

**File S1. Sub-networks 1 – 9 and Their Composite Outcomes**

**Sub-network 1 and its composite outcomes**

The sub-network 1 includes 46 nodes and 969 edges (Figure S1). The score of cluster density is 21.065, which indicates that sub-network 1 is the most densely connected because it possesses the highest cluster density score among 9 sub-networks. In Figure S1, the dark red nodes represent higher network density based on MCODE. Dark green edges represent very small p-values. There are 31 nodes that represent up-regulated, eight down-regulated, and seven differently regulated. Most gene lists (67.39%) involving up-regulated nodes are related to seven biological themes and 25 treatments or conditions.

Table S1 shows composite outcomes. There are 46 gene lists and 256 most frequently shared genes identified in this sub-network. They are regulated by 32 treatments or conditions from 32 publications related to 11 biological themes involving development, metabolism, disease, yield, function, genome analysis, immune, pathogen, mechanism, energy, virus, and photosynthesis in *Arabidopsis*. The top 10 most frequently shared genes with their gene descriptions were specifically listed corresponding to different gene lists, different biological themes, and treatments or conditions. These genes are the most active in sub-network 1. For example, gene AT4G14365 (“putative E3 ubiquitin-protein ligase XBAT34”) has the highest frequency of 35, which indicates it is the most active gene because it is regulated simultaneously under 35 gene lists in sub-network 1. In other words, the gene connects directly 35 gene lists. Therefore, it is the strongest link in sub-network 1.

The most significant function of sub-network 1 is biological process in response to chitin (i.e. the most enriched term in sub-network 1 is “response to chitin”) based on results of analysis of DAVID (Table 1). This indicates sub-network 1 is specifically associated with the chitin signaling pathway rather than by random chance. The other significant functions of sub-network 1 are responding to carbohydrate stimulus, organic substance, defense response, and bacterium based on the analysis of DAVID with a cutoff of 1.60E-16 p-value. This suggests that sub-network 1 involves multiple signaling pathways.


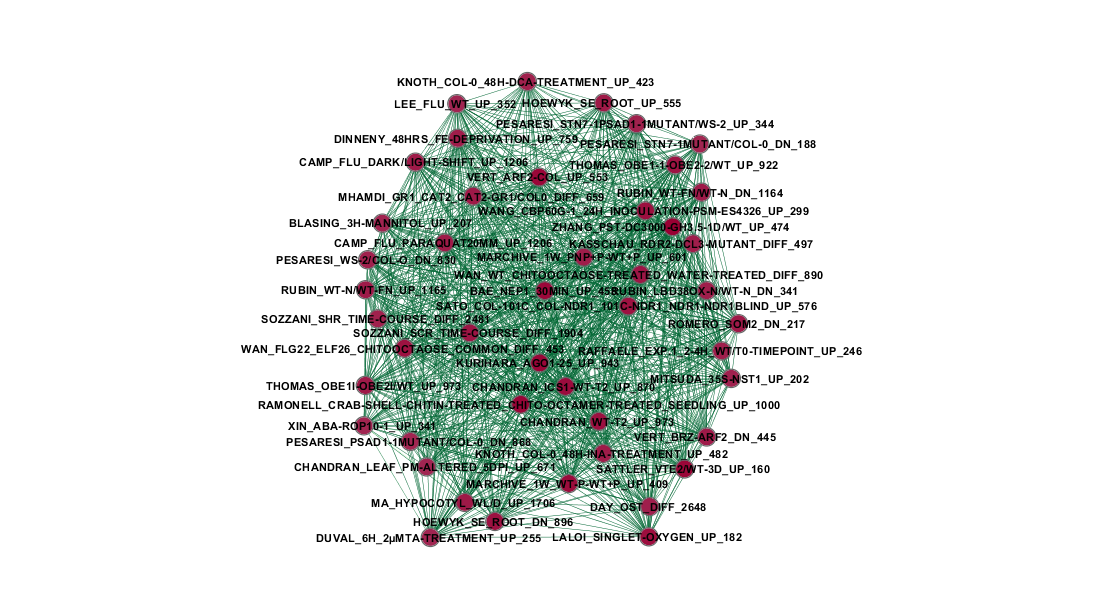


**Figure S1.** Sub-network 1 corresponding to cluster 1. Node = name of gene list. Node Color = MCODE_Scores from small to large and corresponds to color from light green to dark red. Edge Color = p-values from large to small and corresponds to color from grey to dark green. (Figure S1 is the same as Figure 3).

**Table S1.** Results of sub-network 1 corresponding to cluster 1.

**Table S1.** (Continued)

**Table S1.** (Continued)

*Note: G1 = AT4G14365 (putative E3 ubiquitin-protein ligase XBAT34).

G2 = AT5G39670 (putative calcium-binding protein CML45). G3 = AT3G50930 (cytochrome BC1 synthesis). G4 = AT1G27730 (zinc finger protein STZ/ZAT10).

G5 = AT4G39670 (glycolipid transfer protein). G6 = AT2G22500 (uncoupling protein 5). G7 = AT5G26920 (Cam-binding protein 60-like G). G8 = AT5G52760 (copper transport family protein). G9 = AT1G19020 (hypothetical protein). G10 = AT4G33050 (calmodulin-binding protein).

**Sub-network 2 and its composite outcomes**

Sub-network 2 is shown in Figure. S2 and Table S2. It includes 54 nodes (gene lists) and 168 most frequently shared genes, which are regulated under 38 different treatments or conditions from 38 publications related to 10 biological themes. The score of cluster density is 9.907. There are 17 nodes to be up-regulated, 34 to be down-regulated, and three to be differently regulated. Most gene lists (62.96%) involving down-regulated nodes are related to nine biological themes and 23 treatments or conditions. Compared to sub-network 1, sub-network 2 has a lower cluster density score with even more treatments or conditions. Nine themes in sub-network 2 are common with sub-network 1: development, disease, function, genome analysis, mechanism, metabolism, photosynthesis, virus, and yield. This indicates the two sub-networks have relationships linked by same themes.

More interestingly, no genes is common between the 256 most frequently shared genes in sub-network 1 and the 168 most frequently shared genes in sub-network 2. This indicates the two sub-networks have relatively independent functions. The most significant function of sub-network 2 is biological process of plastid thylakoid membrane based on results of DAVID (Table 1), suggesting that sub-network 2 is specifically associated with plastid thylakoid membrane, i.e. the lipid bilayer membrane of any thylakoid within a plastid. The other significant functions of sub-network 2 are response to chloroplast thylakoid membrane, thylakoid membrane, plastid thylakoid, and chloroplast thylakoid based on DAVID with cut-off p-value of 6.8E-58. The 10 most frequently shared genes with their gene descriptions corresponding to gene lists, biological themes, and treatments or conditions were specifically listed in Table 4. Gene AT4G27030 (fatty acid desaturase A), for example, has the highest frequency of 20, indicating it is the most active gene in sub-network 2.


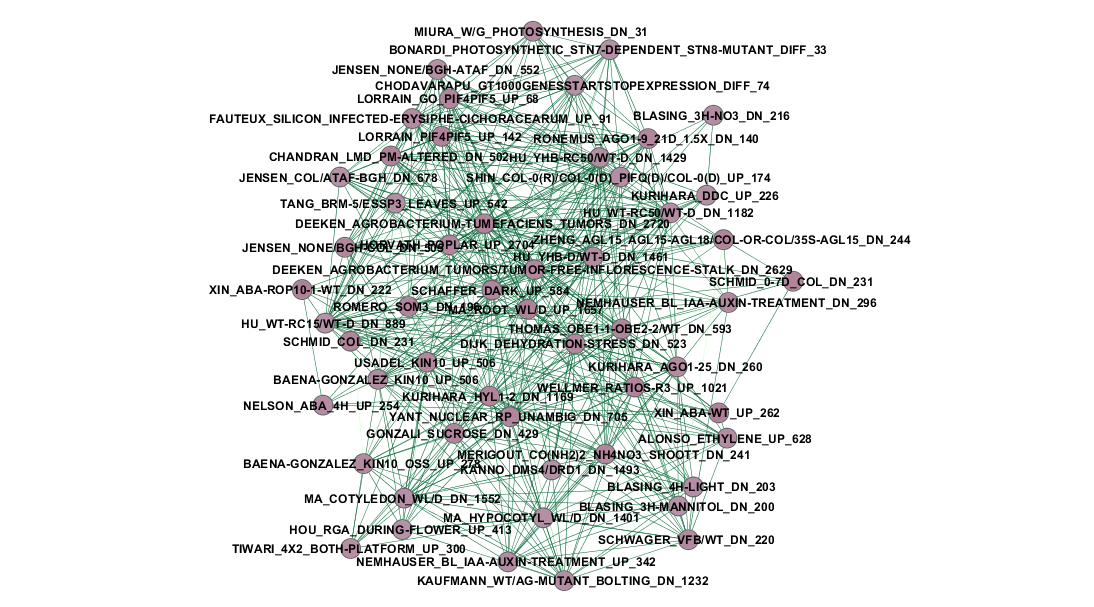


**Figure S2.** Sub-network 2 corresponding to cluster 2. Node = name of gene list. Node Color = MCODE_Scores from small to large and corresponds to color from light green to dark red. Edge Color = p-values from large to small and corresponds to color from grey to dark green. (Figure S2 is the same as Figure 4).

**Table S2.** Results of sub-network 2 corresponding to cluster 2.

**Table S2.** (Continued)

**Table S2.** (Continued)

*Note: G1 = AT4G27030 (fatty acid desaturase A). G2 = AT1G29660 (GDSL esterase/lipase). G3 = AT2G21330 (fructose-bisphosphate aldolase, class I).

G4 = AT3G01500 (carbonic anhydrase 1). G5 = AT4G37980 (cinnamyl alcohol dehydrogenase 7). G6 = AT1G06680 (oxygen-evolving enhancer protein 2-1).

G7 = AT1G54040 (epithiospecifier protein). G8 = AT1G71030 (myb proto-oncogene protein). G9 = AT3G16250 (NDH-dependent cyclic electron flow 1). G10 = AT3G50820 (oxygen-evolving enhancer protein 1-2).

**Sub-network 3 and its composite outcomes**

The sub-network 3 includes 33 nodes (gene lists) and 124 most frequently shared genes (Figure S3 and Table S3). There are 28 nodes to be up-regulated, four to be down-regulated, and one to be differently regulated. Most gene lists (84.85%) involving up-regulated nodes are related to nine biological themes and 20 treatments or conditions. By contrast to sub-networks 1 and 2, sub-network 3 is smaller in size, has a lower cluster density score, and less treatments or conditions for gene lists. Sub-network 3 are regulated by 25 treatments or conditions from 25 publications associated with 11 biological themes. Nine themes in sub-network 3 are common with sub-network 1: development, disease, energy, function, immune, mechanism, metabolism, photosynthesis, and virus. There are seven common themes (development, disease, function, mechanism, metabolism, photosynthesis, and virus) between sub-networks 3 and 2. These indicate sub-networks 3 and 1 or 2 have relationships linked by the same themes.

The 10 most frequently shared genes with their gene descriptions corresponding to each gene list in sub-network 3 are specifically listed in Table 5. Gene AT2G18690 has the highest frequency at 17, indicating it is the most active genes (the strongest link) in sub-network 3. Furthermore, the most frequently shared genes in sub-network 3 have lower frequency, which means they are less active than those in sub-networks 1 and 2. Interestingly, there is no common gene between the 168 most frequently shared genes in sub-network 2 and the 124 most frequently shared genes in sub-network 3. There are 72 genes of intersection between sub-networks 1 and 3. This indicates sub-networks 2 and 3 have relatively independent functions and sub-networks 1 and 3 maybe are similar to functions. The most significant function of sub-network 3 is biological process in response to chitin based on results of analysis of DAVID. This indicates sub-network 3 is specifically associated with the chitin signaling pathway, which is the same as sub-network 1 but is different from sub-networks 2 (Table 1).

**
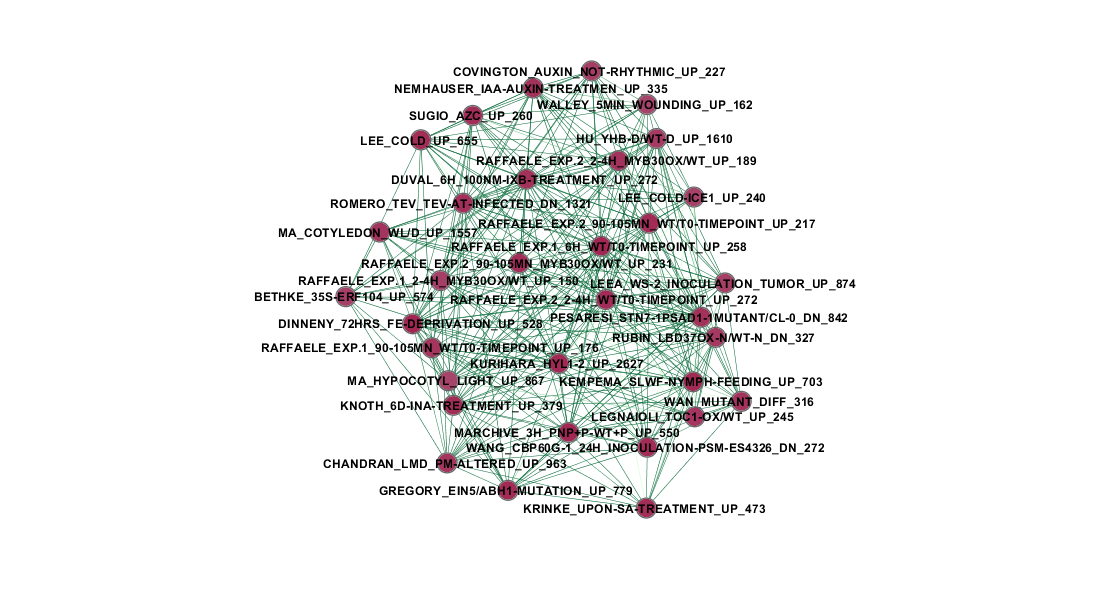
**

**Figure S3.** Sub-network 3 corresponding to cluster 3. Node = name of gene list. Node Color = MCODE_Scores from small to large and corresponds to color from light green to dark red. Edge Color = p-values from large to small and corresponds to color from grey to dark green. Edge label = number of overlapping genes between both nodes. (Figure S3 is the same as Figure 5).

**Table S3.** Results of sub-network 3 corresponding to cluster 3.

**Table S3.** (Continued)

*Note: G1 = AT2G18690 (hypothetical protein). G2 = AT2G46400 (putative WRKY transcription factor 46). G3 = AT5G59820 (C2H2-type zinc finger protein).

G4 = AT1G19180 (protein TIFY 10A). G5 = AT1G27730 (zinc finger protein STZ/ZAT10). G6 = AT2G22500 (uncoupling protein 5). G7 = AT2G43570

(chitinase class 4-like protein). G8 = AT3G18830 (Polyol transporter 5).

G9 = AT3G26830 (cytochrome P450 71B15). G10 = AT1G43910 (P-loop containing nucleoside triphosphate hydrolase-like protein).

**Sub-network 4 and its composite outcomes**

The sub-network 4 corresponding to cluster 4/module 4 is shown in Figure S4 and Table S4. It includes 48 gene lists and 155 most frequently shared genes that were regulated by 32 treatments or conditions related to 11 biological themes. The top 10 most frequently shared genes were specifically listed. Gene AT1G74670 (“putative gibberellin-regulated protein”) has the highest frequency of 18. It is the most active genes (the strongest links) in sub-network 4. The most enriched term is “response to auxin stimulus” based on DAVID, which indicates sub-network 4 is specifically associated with auxin stimulus signaling pathway rather than by random chance.


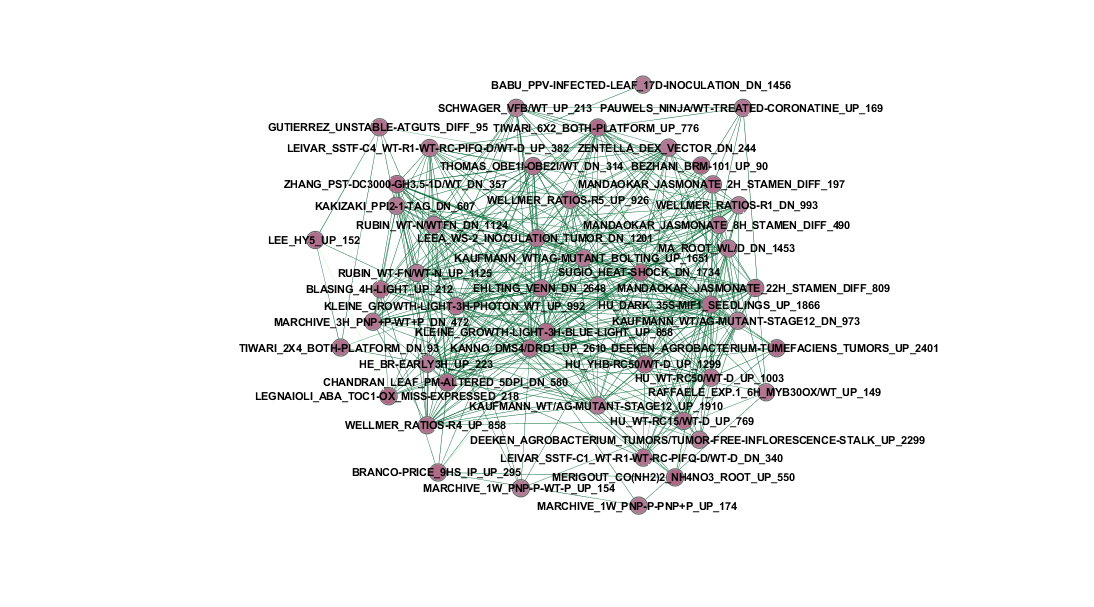


**Figure** S4**.** The sub-network 4 correspoding to cluster 4. Node = name of gene list. Node Color = MCODE_Scores from small to large and corresponds to color from light green to dark red. Edge Color = p-values from large to small and corresponds to color from grey to dark green. Edge label = number of overlapping genes between both nodes.

**Table** S4**.** Results of sub-network 4 corresponding to cluster 4.

**Table** S4 **(Continued)**

*Note: G1-G10 = AT1G74670, AT1G04240, AT1G69530, AT5G44680, AT2G21880, AT2G44740, AT3G45140, AT4G34760, AT4G38840, and AT5G47240.

**Sub-network 5 and its composite outcomes**

The sub-network 5 corresponding to cluster 5/module 5 is shown in Figure S5 and Table S5. It includes 34 nodes (gene lists) and 109 most frequently shared genes, which are regulated by 26 treatments or conditions related to 11 biological themes. The top 10 most frequently shared genes are specifically listed corresponding to different gene lists, biological themes, and treatments or conditions. They are the most active genes in sub-network 5. Gene AT1G29500 (“SAUR-like auxin-responsive protein”) has the highest frequency of 10, which means the gene is the strongest link in sub-network 5. The most enriched term in sub-network 5 is cell wall based on functional analysis of DAVID. This indicates sub-network 5 is specifically associated with cell wall signaling pathway.

**
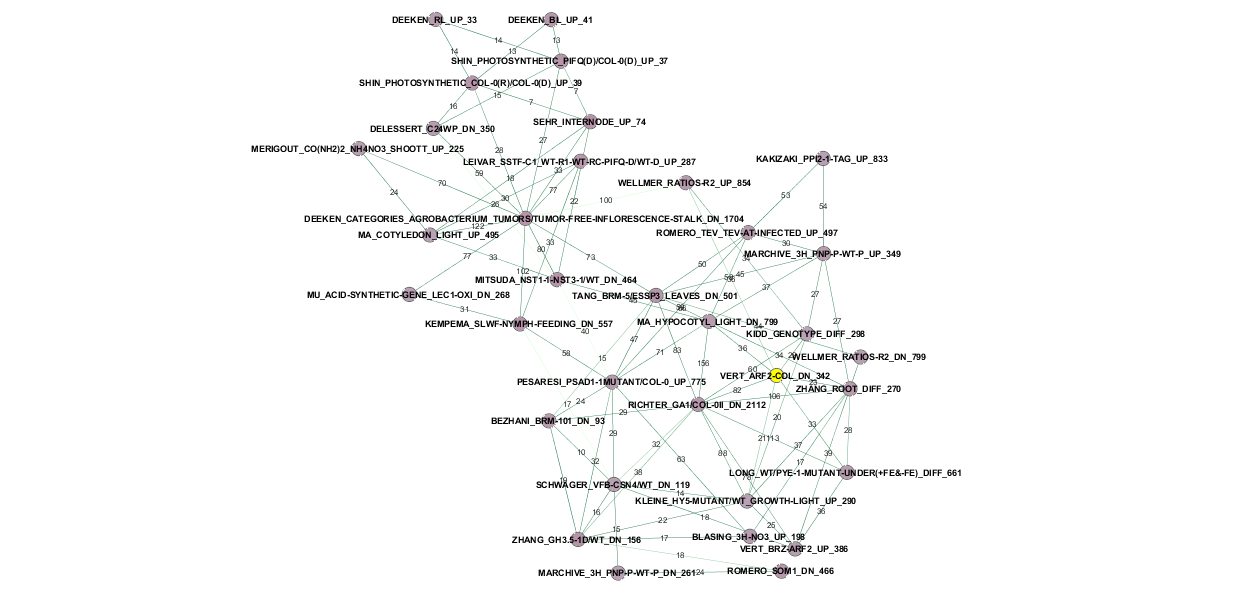
**

**Figure** S5**.** The sub-network 5 corresponding to Cluster 5. Node = name of gene list. Node Color = MCODE_Scores from small to large and corresponds to color from light green to dark red. Edge Color = p-values from large to small and corresponds to color from grey to dark green. Edge label = number of overlapping genes between both nodes.

**Table** S5**.** Results of sub-network 5 corresponding to cluster 5.

**Table** S5 **(continued)**

*Note: G1-G10 = AT1G29500, AT2G16060, AT1G08380, AT1G30380, AT1G33811, AT1G67865, AT1G72610, AT2G02130, AT2G21650, and AT4G28750.

**Sub-network 6 and its composite outcomes**

The sub-network 6 corresponding to cluster 6/module 6 is shown in Figure S6 and Table S6. It includes 9 nodes (gene lists) and 34 most frequently shared genes, which are regulated by 6 treatments or conditions from 6 publications related to 3 biological themes. The top 10 most frequently shared genes are specifically listed corresponding to different gene lists, biological themes, and treatments or conditions. Gene AT1G02790 (“polygalacturonase 4”) has the highest frequency at 10. They are the most active genes (the strongest links) in sub-network 6.

The most enriched term in sub-network 6 is external encapsulating structure organization based on functional analysis of DAVID. This indicates sub-network 6 is specifically associated with external encapsulating structure organization signaling pathway.


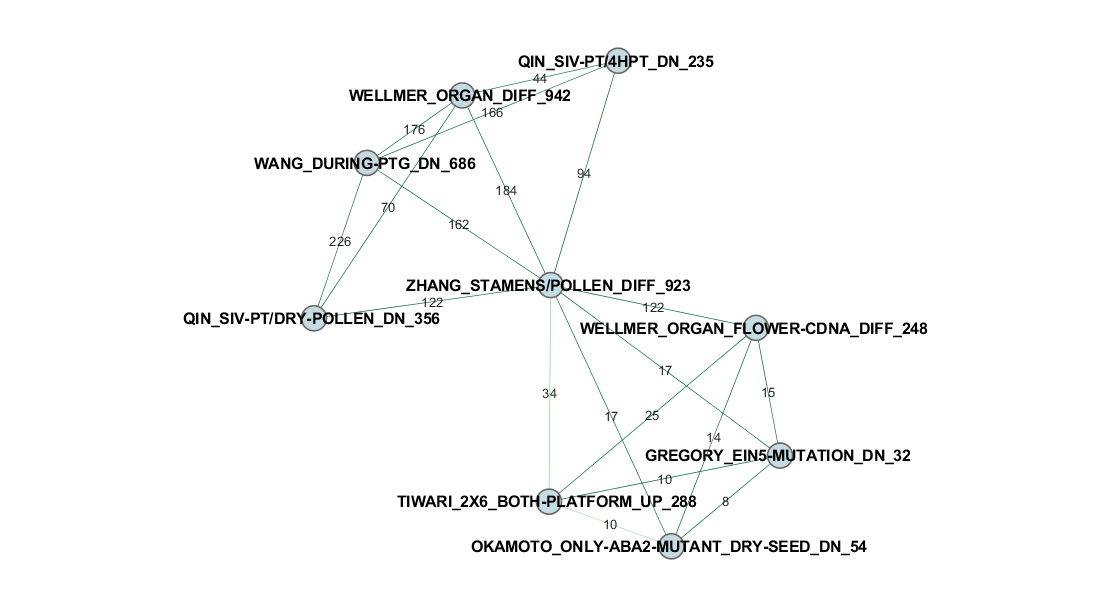


**Figure** S6**.** The sub-network 6 corresponding to cluster 6. Node = name of gene list. Node Color = MCODE_Scores from small to large and corresponds to color from light green to dark red. Edge Color = p-values from large to small and corresponds to color from grey to dark green. Edge label = number of overlapping genes between both nodes.

**Table** S6**.** Results of sub-network 6 corresponding to cluster 6.

*Note: G1-G10 = AT1G02790, AT1G54070, AT2G16730, AT2G26850, AT2G47050, AT3G07820, AT3G07850, AT3G28830, AT4G16745, and AT1G13950.

**Sub-network 7 and its composite outcomes**

The sub-network 7 corresponding to cluster 7/module 7 is shown in Figure S7 and Table S7. It includes 15 nodes (gene lists) and produces 53 most frequently shared genes that were regulated by 14 treatments or conditions from 14 publications related to 6 biological themes. The top 10 most frequently shared genes corresponding to different gene lists, biological themes, and treatments were specifically listed. Gene AT1G73260 (“kunitz trypsin inhibitor 1”) has the highest frequency of 8. They are the most active genes (the strongest links) in sub-network 7. The most enriched term in sub-network 7 is glycoside biosynthetic process based on functional analysis of DAVID. This indicates sub-network 7 is specifically associated with glycoside biosynthetic process signaling pathway.


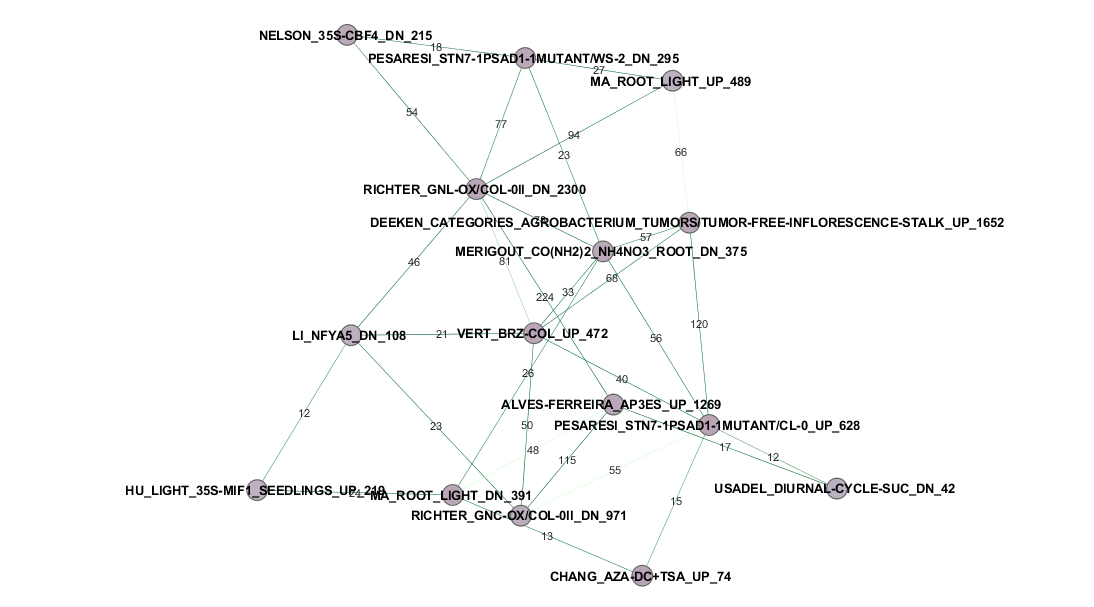


**Figure** S7**.** The sub-network 7 corresponding to cluster 7. Node = name of gene list. Node Color = MCODE_Scores from small to large and corresponds to color from light green to dark red. Edge Color = p-values from large to small and corresponds to color from grey to dark green. Edge label = number of overlapping genes between both nodes.

**Table** S7**.** Results of sub-network 7 corresponding to cluster 7.

*Note: G1-G10 = AT1G73260, AT2G30600, AT1G19530, AT1G64660, AT2G18700, AT2G38530, AT3G16450, AT4G04830, AT4G11650, and AT4G13770.

**Sub-network 8 and its composite outcomes**

The sub-network 8 corresponding to cluster 8/module 8 is shown in Figure S8 and Table S8. It includes 6 gene lists and 66 most frequently shared genes, which were regulated by 5 treatments or conditions from 5 publications related to 5 biological themes. The top 10 most frequently shared genes were specifically listed corresponding to different gene lists, biological themes, and treatments or conditions. Gene AT1G56110 (“homolog of nucleolar protein NOP56”) has the highest frequency of 6. They are the most active genes (the strongest links) in sub-network 8. The most enriched term in sub-network 8 is membrane-enclosed lumen based on functional analysis of DAVID. This indicates sub-network 8 is specifically associated with membrane-enclosed lumen signaling pathway.

**
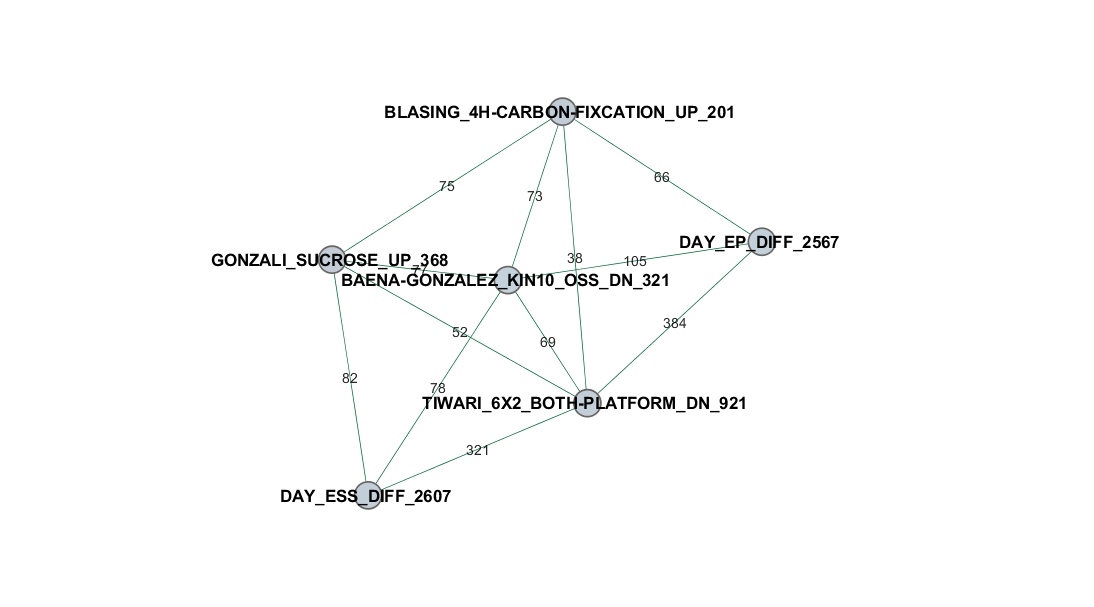
**

**Figure** S8**.** The sub-network 8 corresponding to cluster 8. Node = name of gene list. Node Color = MCODE_Scores from small to large and corresponds to color from light green to dark red. Edge Color = p-values from large to small and corresponds to color from grey to dark green. Edge label = number of overlapping genes between both nodes.

**Table** S8**.** Results of sub-network 8 corresponding to cluster 8.

*Note: G1-G10 = AT1G56110, AT3G05060, AT3G44750, AT1G07370, AT1G44900, AT1G55900, AT3G12270, AT3G20330, AT3G23940, and AT3G55010.

**Sub-network 9 and its composite outcomes**

The sub-network 9 including 13 gene lists corresponding to cluster 9/module 9 is shown in Figure S9 and Table S9. It includes 23 most frequently shared genes, which were regulated by 12 treatments or conditions from 12 publications related to 7 biological themes. The top 10 most frequently shared genes were specifically listed corresponding to different gene lists, biological themes, and treatments or conditions. Gene AT1G01470 (“putative desiccation-related protein LEA14”) has the highest frequency of 6. They are the most active genes (the strongest links) in sub-network 9. The most enriched term in sub-network 9 is response to abiotic stimulus based on functional analysis of DAVID. This indicates sub-network 9 is specifically associated with response to abiotic stimulus signaling pathway.


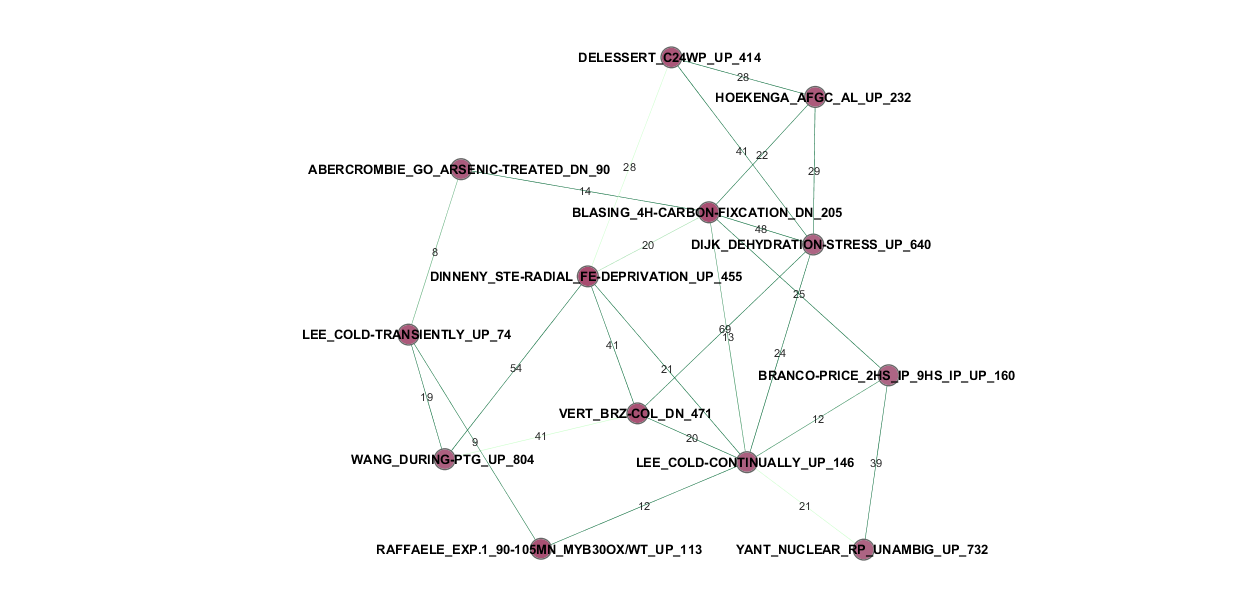


**Figure** S9**.** The sub-network 9 corresponding to cluster 9. Node = name of gene list. Node Color = MCODE_Scores from small to large and corresponds to color from light green to dark red. Edge Color = p-values from large to small and corresponds to color from grey to dark green. Edge label = number of overlapping genes between both nodes.

**Table** S9**.** Results of sub-network 9 corresponding to cluster 9.

*Note: G1-G10 = AT1G01470, AT2G22500, AT2G23810, AT5G05410, AT5G58070, AT5G59820, AT1G09070, AT1G19180, AT1G20440, and AT1G25400.
